# Intra-breath arterial oxygen oscillations detected by a fast oxygen sensor in an animal model of acute respiratory distress syndrome

**DOI:** 10.1093/bja/aeu407

**Published:** 2015-01-28

**Authors:** F. Formenti, R. Chen, H. McPeak, P. J. Murison, M. Matejovic, C. E. W. Hahn, A. D. Farmery

**Affiliations:** 1 Nuffield Department of Clinical Neurosciences, Nuffield Division of Anaesthetics, University of Oxford, Oxford, UK; 2 School of Veterinary Sciences, University of Bristol, Bristol, UK; 3 Biomedical Centre, Charles University in Prague, Faculty of Medicine in Pilsen, alej Svobody 80, 304 60 Pilsen, Czech Republic; 4 First Medical Department, Charles University in Prague, Faculty of Medicine in Pilsen, alej Svobody 80, 304 60 Pilsen, Czech Republic

**Keywords:** acute respiratory distress syndrome, arterial oxygen monitoring, cyclical atelectasis, fibreoptic sensor

## Abstract

**Background:**

There is considerable interest in oxygen partial pressure (*P*o_2_) monitoring in physiology, and in tracking *P*o_2_ changes dynamically when it varies rapidly. For example, arterial *P*o_2_ ($P{\text{a}}_{O_{2}}$) can vary within the respiratory cycle in cyclical atelectasis (CA), where $P{\text{a}}_{O_{2}}$ is thought to increase and decrease during inspiration and expiration, respectively. A sensor that detects these $P{\text{a}}_{O_{2}}$ oscillations could become a useful diagnostic tool of CA during acute respiratory distress syndrome (ARDS).

**Methods:**

We developed a fibreoptic *P*o_2_ sensor (<200 µm diameter), suitable for human use, that has a fast response time, and can measure *P*o_2_ continuously in blood. By altering the inspired fraction of oxygen ($FI_{O_{2}}$) from 21 to 100% in four healthy animal models, we determined the linearity of the sensor's signal over a wide range of $P{\text{a}}_{O_{2}}$ values *in vivo*. We also hypothesized that the sensor could measure rapid intra-breath $P{\text{a}}_{O_{2}}$ oscillations in a large animal model of ARDS.

**Results:**

In the healthy animal models, $P{\text{a}}_{O_{2}}$ responses to changes in $FI_{O_{2}}$ were in agreement with conventional intermittent blood-gas analysis (*n*=39) for a wide range of $P{\text{a}}_{O_{2}}$ values, from 10 to 73 kPa. In the animal lavage model of CA, the sensor detected $P{\text{a}}_{O_{2}}$ oscillations, also at clinically relevant $P{\text{a}}_{O_{2}}$ levels close to 9 kPa.

**Conclusions:**

We conclude that these fibreoptic $P{\text{a}}_{O_{2}}$ sensors have the potential to become a diagnostic tool for CA in ARDS.

**Editor's key points**

- A fibreoptic sensor was developed which can measure *P*o_2_ continuously in blood.
- The sensor was tested in animal models over a range of $P{\text{a}}_{O_{2}}$ values.
- The sensor agreed with conventional blood gas analysis.
- In a model of cyclical atelectasis, the sensor detected arterial *P*o_2_ oscillations.
- The sensor has clinical potential.

It is commonly accepted that arterial oxygenation remains essentially constant during the respiratory cycle in the healthy lung. Only minimal variations have been observed, with a period corresponding to the breathing period.^1–4^ In contrast, in some conditions of lung injury, the arterial partial pressure of oxygen ($P{\text{a}}_{O_{2}}$) is known to oscillate to a great extent.^5–7^ It is thought that these $P{\text{a}}_{O_{2}}$ oscillations are caused by alveolar opening during inspiration, when $P{\text{a}}_{O_{2}}$ increases, and by alveolar collapse during expiration, when $P{\text{a}}_{O_{2}}$ decreases; this phenomenon is called cyclical atelectasis (CA). This understanding is supported in part by evidence from imaging studies using, for example, computed tomography and positron emission tomography scans (reviewed in^8^ and^9^) and in part by studies using optical intravascular sensors, which showed $P{\text{a}}_{O_{2}}$ oscillations in animal models of lung injury.^6,7,10–13^

These insightful investigations have furthered our understanding of the relationship between $P{\text{a}}_{O_{2}}$ levels and lung mechanics, and have better informed the management of mechanical ventilation. However, investigations using fast $P{\text{a}}_{O_{2}}$ sensors have not yet been conducted in human patients because early embodiments of these sensors contained ruthenium, a toxic material,^14^ which needs to be exposed in order for the sensors to achieve a sufficiently rapid response time.^15^ The assessment of individual sensors is a critical step when using these prototype sensors for physiological or medical research.^15–17^ Furthermore, the relevance of animal studies and their translation into medical practice remain unclear, due, for example, to anatomical and pathophysiological differences between species.

In order to avoid the requirement for a toxic component in these sensors, we produced a $P{\text{a}}_{O_{2}}$ sensor based on a platinum complex, immobilized in a polymer matrix,^18,19^ which has the potential to be safely used in humans, once its reliability is established in large animal models. We recently showed that this sensor can detect up to 60 *P*o_2_ oscillations per minute *in vitro*, simulating the occurrence of CA at a high respiratory rate.^17^ We also showed that, when exposed to non-heparinized blood, the sensor is resistant to blood clotting at least for a period of 24 h *in vivo*.^17^

This sensor has the potential to become a useful diagnostic tool for CA in the acute respiratory distress syndrome (ARDS), or in other clinical scenarios where $P{\text{a}}_{O_{2}}$ is thought to change rapidly. Moreover, the continuous real-time $P{\text{a}}_{O_{2}}$ signal would provide immediate feedback on the effect of changes in ventilatory settings, which could be tailored on an individual basis, possibly leading towards a more personalized form of ventilation therapy.

This study aimed at establishing the sensor's response time, exploring the $P{\text{a}}_{O_{2}}$ range over which the sensor can generate reliable results, using standard blood gas analysis as a control measurement, and assessing whether it could detect $P{\text{a}}_{O_{2}}$ oscillations in an ovine model of ARDS *in vivo*.

## Methods

The methods for the assessment of sensor's response time *in vitro* are presented elsewhere,^18,20^ and outlined in the Supplementary material.

All the animal experiments conformed to the National Institutes of Health Guidelines for the Use of Laboratory Animals. The protocols for the linearity experiments at the Charles University, Czech Republic, were approved by the local University Animal Care Committee. The protocols for the ovine lavage model studies were approved by the UK Home Office. At the end of each study, the animals were killed under anaesthesia with an overdose of pentobarbital (∼100 mg kg^−1^). Relevant sections of the ARRIVE guidelines were adhered to.

### Assessment of sensor linearity

The *in vivo* linearity of the polymethyl methacrylate (PMMA) sensor was first assessed in experiments at the Faculty of Medicine, Charles University, Plzen, Czech Republic. One female domestic pig (weight 38 kg, age 3 months) was studied during the 8 h recovery and stabilization period from surgery, before the beginning of a separate experiment on the same animal.

Anaesthesia was induced with i.v. propofol (1–2 mg kg^−1^) and ketamine (2 mg kg^−1^). The trachea was intubated and the lungs were mechanically ventilated with tidal volumes of 8 ml kg^−1^, with a PEEP of 6 cm H_2_O, and $FI_{O_{2}}$ of 35%. The respiratory rate was adjusted to maintain normocapnia. Anaesthesia was maintained with continuous infusions of propofol (1–4 mg kg^−1^ h^−1^) plus fentanyl (10–15 µg kg^−1^ h^−1^). After preparation, the infusion of fentanyl was decreased to 5 µg kg^−1^ h^−1^, and maintained until the end of the experiment. Adequacy of anaesthesia was determined by end tidal agent monitoring, the absence of movement, haemodynamic monitoring (heart rate and arterial pressure), and absence of reflexes. Continuous infusion of Ringerfundin™ solution (Braun Melsungen Ag, Melsungen, Germany) was used as a fluid replacement in doses of 10 ml kg^−1^ h^−1^ during the preparation and reduced to 7 ml kg^−1^ h^−1^ thereafter.

The PMMA sensor was connected to the phase measurement system,^17^ and inserted in the femoral artery through a standard catheter (length 10 cm, diameter 1.2 mm) to monitor $P{\text{a}}_{O_{2}}$ responses to changes in $FI_{O_{2}}$. This was temporarily decreased to 21%, and then increased to 60%, 80%, and 100%, interspersed with recovery periods where $FI_{O_{2}}$ was returned to baseline (i.e. 35%); each condition lasted for about 4 min. The sensor was calibrated *a posteriori*, on the basis of the smallest and greatest $P{\text{a}}_{O_{2}}$ values, as recorded with standard analysis of arterial blood gas (ABG, ABL710, Radiometer, Copenhagen, Denmark). Arterial blood samples were obtained once $FI_{O_{2}}$ had been maintained at 21%, 35%, 60%, 80%, 100%, and 35% for ∼3 min, just before $FI_{O_{2}}$ was changed to a different concentration; ABG results were used as a means of comparison with data continuously recorded through the sensor. The sampling rate was set at 10 Hz.

In a second set of experiments, the linearity of the sensor was assessed at the University of Bristol, School of Veterinary Sciences, where a series of $FI_{O_{2}}$ changes (similar to those presented above) were applied before induction of lung injury in three ovine models. Female sheep [weight 69 (2) kg; age ∼18 months] were obtained from a local commercial farm source. For these studies, the sensor was calibrated *in vivo*, on the basis of standard ABG analysis at baseline (i.e. $FI_{O_{2}}$=21%), and at $FI_{O_{2}}$ of 35%; this range of values was chosen because it was associated with $P{\text{a}}_{O_{2}}$ values of about 11–20 kPa, where ABG analysis is frequently used. One PMMA sensor was used in each *in vivo* study (i.e. one for the porcine model, and three for the ovine models, such that a total of four sensors was used).

### Detection of within-breath oscillations

To determine whether the sensor was capable of detecting within-breath $P{\text{a}}_{O_{2}}$ oscillations, one sheep with lavage-induced lung injury was studied at the School of Veterinary Sciences, University of Bristol, UK. An 18 G catheter was placed in the right cephalic vein and anaesthesia was induced with i.v. midazolam (0.4 mg kg^−1^) and propofol to effect (∼2 mg kg^−1^). The trachea was intubated and anaesthesia maintained with isoflurane (∼1% end-tidal) with the Anaconda system (Sedana Medical AB, Stockholm, Sweden). The sheep was positioned in sternal recumbency, morphine (0.15 mg kg^−1^ i.v.) was administered together with remifentanil or alfentanil infusion. Hartmann's solution was infused i.v. at ∼10 ml kg^−1^ h^−1^. Adequacy of anaesthesia was determined by end tidal agent monitoring, the absence of movement, haemodynamic monitoring (heart rate and arterial pressure), and absence of reflexes. A catheter was placed in an auricular artery for arterial pressure measurement. The ECG, direct arterial pressure, $S{\text{p}}_{O_{2}}$, and end-tidal carbon dioxide were monitored throughout. The PMMA sensor was positioned in the carotid artery via a standard arterial catheter (length 8 cm, diameter 0.9 mm), the insertion of which was guided with ultrasound imaging. Ventilation was achieved with a Siemens Servo 900C mechanical ventilator in the pressure-control mode, and adjusted to maintain normocapnia (arterial carbon dioxide tension 4–5 kPa). At this proof-of-concept stage, the respiratory rate was set between 5 and 6 bpm for the detection of $P{\text{a}}_{O_{2}}$ oscillations, in agreement with previous studies.^6,11,12,21^ Although this respiratory rate is not clinically relevant, it was chosen in order to provoke the maximum cyclic recruitment and derecruitment, and hence potentially the greatest $P{\text{a}}_{O_{2}}$ oscillations.^11,22^ Physiological variables were continuously monitored with standard patient monitors (Datex Ohmeda Capnomac Ultima; multi-parameter patient monitor: Datex AS3). Following baseline measurements, warm saline lavages were performed (30 ml kg^−1^ 0.9% NaCl at about 38°C) until a $P{\text{a}}_{O_{2}}/FI_{O_{2}}$ ratio of <13 kPa (98 mm Hg) was achieved, as recorded by standard arterial blood gas analysis.

### Statistical analysis

For the linearity tests, $P{\text{a}}_{O_{2}}$ values were averaged over the relevant 2 s periods, and compared with the associated ABG results. Results were assessed statistically using analysis of variance (IBM SPSS Statistics for Windows, Version 20.0; Armonk, NY, USA). Comparison between results from standard ABG results and from our sensor was performed with the Bland–Altman analysis.^23–25^

## Results

Results from the response time of the sensor *in vitro* experiments are presented in the Supplementary material.

**Fig 1:**
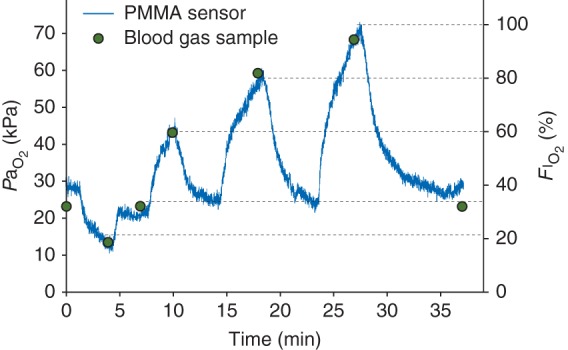
Arterial *P*o_2_ responses to $FI_{O_{2}}$ step changes in one anaesthetized animal (pig) are plotted against time (sampling rate=10 Hz). The blue line shows $P{\text{a}}_{O_{2}}$ recorded by the PMMA sensor, the circles show results from arterial blood gas sampling at 0, 4, 7, 10, 18, 27, and 37 minutes; $FI_{O_{2}}$ at these time points was 21, 35, 60, 80, 100, and 35% respectively. The dotted horizontal lines highlight the $FI_{O_{2}}$ values at the time when blood samples were taken.

**Fig 2:**
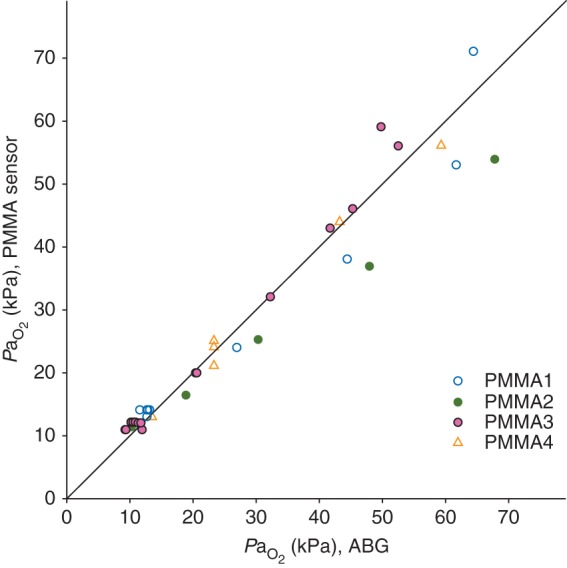
PMMA sensor $P{\text{a}}_{O_{2}}$ results (average value for 2 s) plotted against standard ABG analysis results (*n*=39). Sensors coded PMMA1, PMMA2, and PMMA3 were used in the ovine models; sensor PMMA4 was used in the porcine model. The identity line illustrates the degree of agreement between ABG analysis and the PMMA sensor.

**Fig 3:**
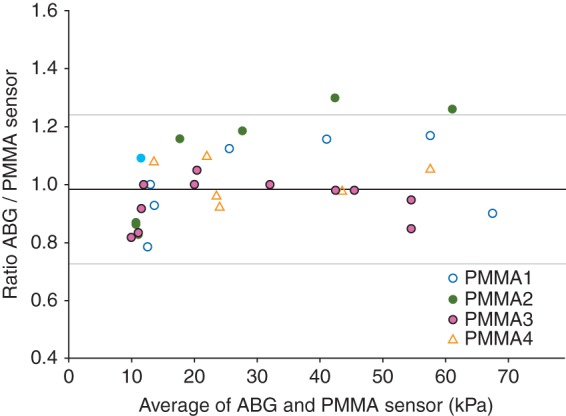
$P{\text{a}}_{O_{2}}$ values measured using ABG and the PMMA sensor (*n*=39) compared by the Bland–Altman plot; the horizontal axis is the paired average, and the vertical axis is the paired ratio (ABG/PMMA). Sensor codes are the same as in given in Figure 2. The middle solid line represents the bias (0.98), and top and bottom grey lines represent the 95% limits of agreement [mean ratio (2 sd); sd was 0.13].

$P{\text{a}}_{O_{2}}$ values recorded continuously in response to changes in $FI_{O_{2}}$ in one anaesthetized, non-heparinized animal (temperature 38°C) and the contemporaneous standard ABG results are shown in Figure 1. The sensor-recorded $P{\text{a}}_{O_{2}}$ values matched those measured by ABG analysis, in a range between 10 and 73 kPa, over a period of 37 min. The agreement between $P{\text{a}}_{O_{2}}$ results obtained with our sensors and results from the standard ABG analysis, as observed during $FI_{O_{2}}$ change experiments, are given in Figure 2. Combining data from ovine and porcine experiments allowed a collection of 39 samples in total. The difference between recorded sensor values and ABG values marginally increased at higher values of $P{\text{a}}_{O_{2}}$. A Bland–Altman analysis is shown in Figure 3, which compares results obtained with the two methods; the bias ratio value appeared small (0.98) with limits of agreement of 0.73 and 1.24.

**Fig 4:**
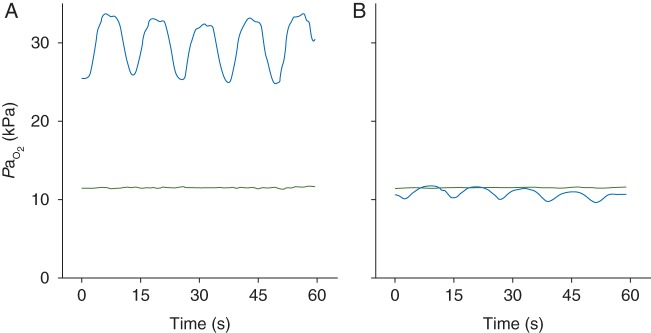
Continuous recordings of $P{\text{a}}_{O_{2}}$ for a period of about 1 min in a healthy ovine lung (green line) and after saline lavages (blue line); the inspired oxygen fraction was 30% and 100%, respectively. $P{\text{a}}_{O_{2}}$ values are shown for (a) mild and (b) severe ARDS-like conditions. Respiratory rate was 5 bpm in each experiment. $P{\text{a}}_{O_{2}}$ oscillations with the same period as breathing were detected in each ARDS model.

Figure 4 shows the results recorded with the PMMA sensor in the healthy lung study [(green lines, inspired fraction of oxygen (F_I_O_2_ = 30%)], where *P*a_O_2__ remained at about 12 kPa over a series of five breaths. Figure 4 also shows results following lung lavage (blue lines, F_I_O_2_ = 100%), where *P*a_O_2__ oscillations with the same period as respiration were detected in (a) mild, and (b) severe ARDS.

## Discussion

This study presents the first *in vivo* applications of a novel, fast intravascular $P{\text{a}}_{O_{2}}$ sensor, showing its capacity to track $P{\text{a}}_{O_{2}}$ changes over a wide range, covering physiological and pathophysiological $P{\text{a}}_{O_{2}}$ levels, and to detect $P{\text{a}}_{O_{2}}$ oscillations associated with the respiratory period at $P{\text{a}}_{O_{2}}$ levels close to 9 kPa.

These prototype sensors are not produced in series, hence differences in performance may vary considerably between sensors.^15,16^ In order to avoid uncertainty in the interpretation of physiological results, it is essential that these sensors are tested individually, and that their performance is clearly defined before they are used for physiological studies. This rationale motivated our *in vitro* tests, where our sensor's response time was sufficiently fast to track the $P{\text{a}}_{O_{2}}$ oscillations that are expected to occur in animal models of lung injury. One limitation of this part of the study was in the testing of the response time of the sensor in the gas phase, while the *in vivo* measurements were performed in the liquid phase (i.e. in whole non-heparinized blood). From a technical perspective, the sensor's response time is independent of the medium that it samples, as it is only a function of oxygen diffusion and solubility within the sensor's matrix. Moreover, we have shown in heparinized whole blood that these sensors are capable of tracking up to 60 $P{\text{a}}_{O_{2}}$ oscillations per minute;^17^ hence, even a relatively slow sensor would be able to follow the oscillations in our *in vivo* study (i.e. about 5 min^−1^). The results of these tests in gas (Supplementary Fig. 1) are presented here for future reference, since it will be easier and cheaper to test other prototype sensors in gas rather than the liquid phase.

The sensor's output is temperature-dependent, so calibration needs to be performed at the temperature at which the sensor will operate. In this study, our sensors were calibrated either *a posteriori* or *in vivo*, when the temperature was known and stable. These forms of calibrations are standard approaches,^22,26,27^ although we acknowledge that further work must be done in order to make these sensors more easily usable for clinical settings, where ‘plug-and-play’ devices are more welcome. In general, a two point calibration is only practically useful if the sensor's signal is linear between these two points. The sensor had a very linear response to $P{\text{a}}_{O_{2}}$ changes, ranging from clinically relevant, low $P{\text{a}}_{O_{2}}$ levels, to rather elevated levels of around 70 kPa; this feature is a technical improvement over currently used optical sensors, which are normally designed to monitor either high or low *P*o_2_ levels. In particular, our sensors were in strong agreement with results from standard blood gas analysis for the lower part of the $P{\text{a}}_{O_{2}}$ range studied, which is the range that is physiologically and clinically most interesting. As oxygen quenches the fluorescence intensity, the PMMA sensor's signal is intrinsically reduced at high *P*o_2_ levels, a technical factor that may have increased the variability of the results and reduced the degree of agreement with standard ABG for $P{\text{a}}_{O_{2}}$ values >40 kPa.

Apart from their accuracy, one of the greatest advantages of these fast sensors is their capacity to record $P{\text{a}}_{O_{2}}$ continuously, giving the opportunity to study dynamic $P{\text{a}}_{O_{2}}$ changes. The preliminary test of the sensor *in vivo* in a large animal model showed that the sensor accurately and rapidly responded to $FI_{O_{2}}$ changes, as confirmed by standard ABG analysis. In response to a rapid change in $FI_{O_{2}}$, the $P{\text{a}}_{O_{2}}$ response appeared to be bi-phasic, characterized by an initial rapid phase, followed by a slower one. Although we can only speculate on the implications of this result, it seems possible that ultrafast $P{\text{a}}_{O_{2}}$ sensors could provide useful information for respiratory physiology and pathophysiology, in particular with reference to ventilatory distribution and oxygen uptake efficiency even in the absence of atelectasis/recruitment.

Fast response time sensors afford the possibility to detect rapid $P{\text{a}}_{O_{2}}$ oscillations within breath in real time, like the oscillations that occur in CA.^6,7^ In this respect, the novelty of our work does not lay at the proof-of-concept level, but rather in its potential to confirm this phenomenon in humans. Most importantly, to the best of our knowledge, this study is the first to show $P{\text{a}}_{O_{2}}$ oscillations in a clinically relevant range; previous studies investigated $P{\text{a}}_{O_{2}}$ levels that are much greater than those observed in a clinical scenario.^6,11,22,26,27^ An alternative approach to assess the degree of variable shunt associated with CA involves standard ABG analysis, where arterial blood is sampled close to the end-inspiratory and end-expiratory stages,^13^ a procedure that is rather cumbersome and, in our experience, unreliable.

The within-breath variation in $P{\text{a}}_{O_{2}}$ levels is thought to be associated with CA, where gas exchange between alveoli and pulmonary circulation is impaired during the expiratory phase of the respiratory cycle, when alveolar collapse occurs.^6,7^ This conclusion is also supported by dynamic computed tomography, positron emission tomography, and electric impedance tomography studies, showing less or more dense lung regions, presumably indicating aerated or collapsed lung.^28–31^ As in many previous studies of $P{\text{a}}_{O_{2}}$ oscillations in models of CA, one of the limitations of our study was in the lack of direct evidence of lung collapse, which was assumed on the basis of intermittently low $P{\text{a}}_{O_{2}}$ values.

The nature of the relationship between the magnitude of $P{\text{a}}_{O_{2}}$ oscillations and CA is complex. It is generally accepted that an increase in the magnitude of $P{\text{a}}_{O_{2}}$ oscillations is associated with greater recruitment and derecruitment in CA. However, these phenomena must be looked at also in consideration of the mean $P{\text{a}}_{O_{2}}$; advanced mathematical modelling characterizing associations and relationships between these variables will contribute to a more accurate clinical interpretation of the oscillations. Studies exploring $P{\text{a}}_{O_{2}}$ oscillations at high mean $P{\text{a}}_{O_{2}}$ levels have the advantage of showing very clear oscillations, larger than the ones shown in this study. This difference in the magnitude of $P{\text{a}}_{O_{2}}$ oscillations is observed most likely because at high mean $P{\text{a}}_{O_{2}}$ levels, the oxygen–haemoglobin dissociation curve is relatively flat, meaning that small changes in the oxygen content of arterial blood (as would result from alveolar recruitment) would change the oxygen partial pressure considerably. Our study shows that these $P{\text{a}}_{O_{2}}$ oscillations are also detectable at a low mean $P{\text{a}}_{O_{2}}$, affording a potential usefulness of the sensor in clinical settings.

In order to test the full potential of these fast sensors in diagnosing CA and in managing mechanical ventilation, further research in large animal models of ARDS is required to facilitate comparison with human adult and paediatric patients. Moreover, whether anatomical and physiological differences between species affect $P{\text{a}}_{O_{2}}$ oscillations in ARDS is unclear, so it is becoming more and more urgent to perform such studies in patients with ARDS, and in mechanically ventilated patients.

## Supplementary material

Supplementary material is available at *British Journal of Anaesthesia* online.

## Authors' contributions

F.F., A.D.F., and C.E.W.H.: conceived and designed the study; F.F., R.C., H.M., A.D.F., M.M., and P.J.M.: performed the experiments; F.F.: performed the data and statistical analysis; F.F.: prepared the figures; F.F., A.D.F., and C.E.W.H.: interpreted the results of experiments; F.F.: drafted the manuscript; F.F., A.D.F., and C.E.W.H.: edited and revised the manuscript; F.F., R.C., M.M., P.J.M, H.M., A.D.F., and C.E.W.H.: approved the final version of the manuscript.

## Declaration of interest

None declared.

## Funding

The laboratory and animal work was supported by a Wellcome Trust Translational Award, Wellcome Trust, UK.

## Supplementary Material

Supplementary Data
