# Supplementary material for: Intra-breath arterial oxygen oscillations detected by a fast oxygen sensor in an animal model of acute respiratory distress syndrome
Source: Br J Anaesth. 2015 Jan 28;114(4):683–8. doi: 10.1093/bja/aeu407 (PMC4364062; doi:10.1093/bja/aeu407)
Supplement: Supplementary Data [file supp_114_4_683__index.html]

Intra-breath arterial oxygen oscillations detected by a fast oxygen sensor in an animal model of acute respiratory distress syndrome — Supplementary Data 

# Intra-breath arterial oxygen oscillations detected by a fast oxygen sensor in an animal model of acute respiratory distress syndrome

## Supplementary Data

Supplementary Data

**Files in this Data Supplement:**

- Supplementary Data - Docx file
