# Supplementary material for: Intra-breath arterial oxygen oscillations detected by a fast oxygen sensor in an animal model of acute respiratory distress syndrome
Source: Br J Anaesth. 2015 Jan 28;114(4):683–8. doi: 10.1093/bja/aeu407 (PMC4364062; doi:10.1093/bja/aeu407)
Supplement: Supplementary Data [file supp_aeu407_aeu407supp.docx]

**Intra-breath arterial oxygen oscillations detected by a fast oxygen sensor in an acute respiratory distress syndrome animal model**

Federico Formenti, Rongsheng Chen, Hanne McPeak , Pamela J. Murison, Martin Matejovic, Clive E. W. Hahn, Andrew D. Farmery

**Supplementary Appendix**

**Methods**

*Assessment of sensor’s response time in vitro*

The procedure for the manufacturing of the PMMA (poly-methyl methacrylate) PO_2_ sensor is presented elsewhere, together with the description of the sensor’s response time testing system [^1^](#_ENREF_1). In the testing chamber, the sensor was exposed to total pressure near-step changes between 100 and 300 kPa, with an associated PO_2_ change between 5 and 15 kPa. A piezoelectric pressure transducer (RS Components Ltd, UK) with a response time of 1 ms was used to determine the response time of the chamber itself, and as a means of comparison. Further details about the sensor and the apparatus have been presented before [^2^](#_ENREF_2). The sensor’s T10-90% response time was calculated as described by Saied *et al.* [^2^](#_ENREF_2).

**Results**

*The sensor detected rapid PO_2_ changes in vitro*

Supplementary Figure 1 shows the absolute pressure near-step change as detected by the piezoelectric transducer (dotted line), and the PO_2_ change detected by the PMMA fibreoptic sensor (solid line). The sensor tested for these *in vitro* experiments was also used later for the detection of PaO_2_ oscillations *in vivo* (results presented in Figure 4 in the main document); all sensors used for other *in vivo* experiments went through this same quality control procedure, and the results presented in Supplementary Figure 1 are representative for other sensors. The sensor’s response time was shown to be about 292 ms, a sufficiently fast response time in order to detect PaO_2_ oscillations at low respiratory rate.


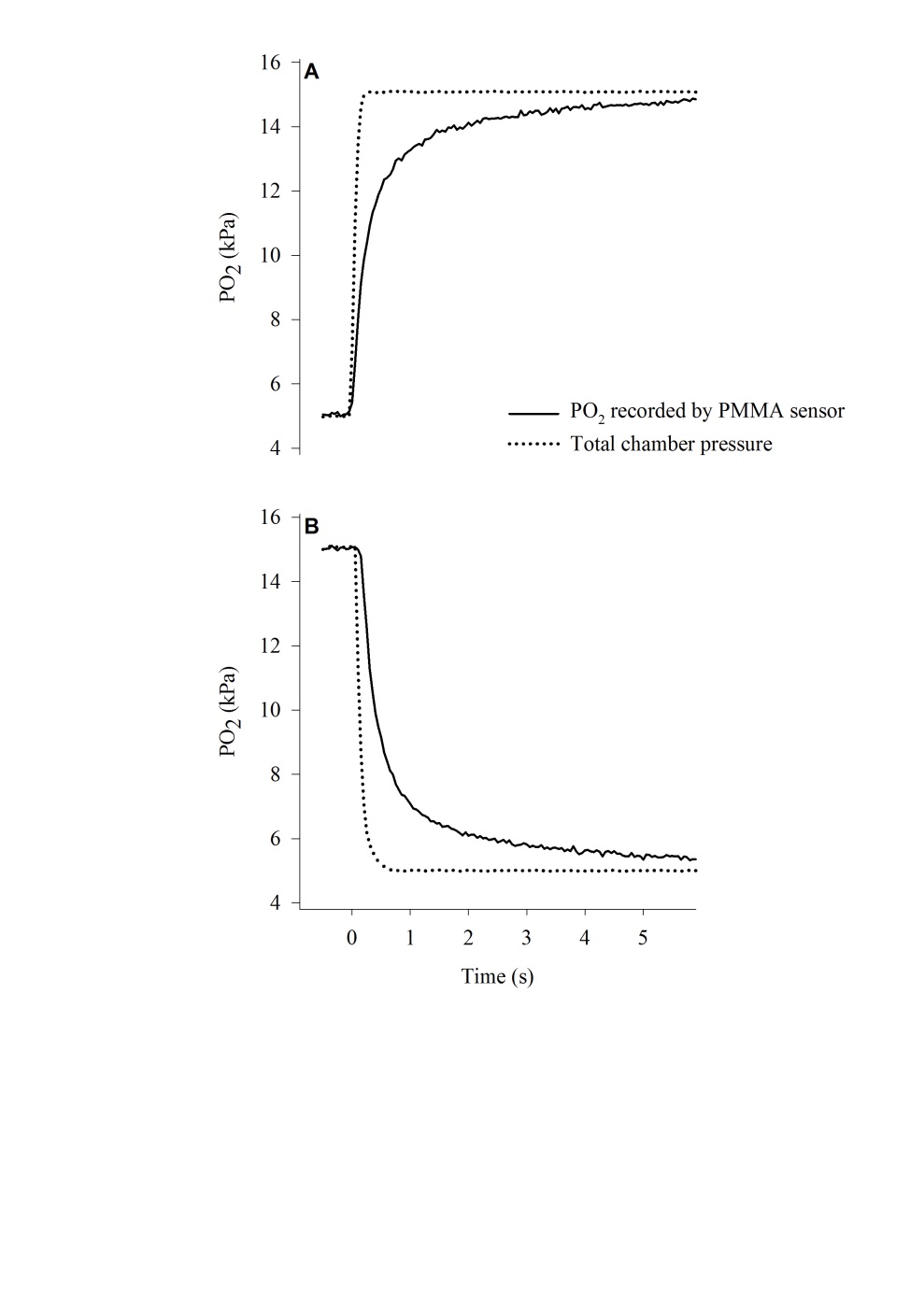


**Supplementary Figure 1**. A near-step change in total gas pressure (hence PO_2_) detected by the piezoelectric pressure transducer (dotted line), and the fibre optic PO_2_ sensor (solid line). (A) Pressure in the chamber was changed between 100 and 300 kPa, with an associated PO_2_ change between 5 and 15 kPa. (B) Pressure drop recorded in the chamber. The sensor’s T10-90% was calculated as 292 ms [^2^](#_ENREF_2).

**References**

1 Chen R, Farmery AD, Obeid A, Hahn CEW. A Cylindrical-Core Fiber-Optic Oxygen Sensor Based on Fluorescence Quenching of a Platinum Complex Immobilized in a Polymer Matrix. *IEEE Sensors Journal* 2012; **12**: 71-5

2 Saied A, Edgington L, Gale L, et al. Design of a test system for fast time response fibre optic oxygen sensors. *Physiol Meas* 2010; **31**: N25-33
